# Large outbreak of *Salmonella* Muenchen linked to dried coconut pieces, September 2020 to July 2021, Germany

**DOI:** 10.1017/S0950268826101423

**Published:** 2026-04-17

**Authors:** Doreen Staat, Sandra Simon, Michael Pietsch, Marina C. Lamparter, Jennie Fischer, Thomas Schewe, Bettina Marlene Rosner, Tobias Wagner, Katja Siling, Judith Overhoff, Christian Schäfers, Olivier Aust, Ekkehard Hiller, Antje Flieger, Klaus Stark, Sofie Gillesberg Lassen, Anika Meinen

**Affiliations:** 1 Department of Infectious Disease Epidemiology, Robert Koch Institute, Germany; 2 Postgraduate Training for Applied Epidemiology (PAE), Robert Koch Institute, Germany; 3 European Programme for Intervention Epidemiology Training (EPIET), European Centre for Disease Prevention and Control, Sweden; 4 National Reference Centre for Salmonella and Other Bacterial Enteric Pathogens, Robert Koch Institute, Germany; 5 Department of Biological Safety, National Reference Laboratory for Salmonella, German Federal Institute for Risk Assessment, Germany; 6 Unit 123 – Crisis Unit Office, Foodborne Outbreaks, Prevention, Federal Office of Consumer Protection and Food Safety Berlin Mitte, Germany; 7 Bavarian Health and Food Safety Authority, Germany; 8 Department Health Promotion Health Protection, Unit Infectious Disease Epidemiology, NRW Centre for Health, Germany; 9 Department of Medical Microbiology, Free and Hanseatic City of Hamburg Institute of Hygiene and Environment, Germany; 10 Chemical and Veterinary Investigation Office Rhein-Ruhr-Wupper (CVUA-RRW), Germany; 11 Chemical and Veterinary Investigation Office Stuttgart, Germany

**Keywords:** Salmonella, outbreak, coconut, case-control study, cgMLST

## Abstract

In September 2020, an unexpected increase in *Salmonella* Muenchen patient isolates and notifications was observed. We investigated the outbreak to identify the vehicle of infection. RKI defined cases as patients with laboratory-confirmed *S.* Muenchen infections reported between September 2020 and July 2021. Genomes of clinical, food, and animal *S.* Muenchen isolates were analysed using cgMLST. We conducted interviews and performed a frequency-matched case–control study. We calculated frequencies and adjusted odds ratios (aOR) using logistic regression. We identified 301 cases in eight federal states in Germany. Hypothesis-generating interviews did not provide a conclusive hint of a possible vehicle. *S.* Muenchen strains were detected in dried coconut pieces, milk powder used for chocolate production, and a wild swan, all with a cgMLST profile indistinguishable from the prominent node comprising 116 patient isolates. Cases included in the case–control study more often consumed dried coconut pieces (22/30) than controls (2/116) (aOR: 176 (95% confidence interval: 32–954)). In this investigation, cgMLST analysis presented identical strains in three different isolate sources. The case–control study supported dried coconut pieces as vehicle of infection demonstrating the importance of interdisciplinary investigations and underscoring the potential impact of unusual vehicles.

## Introduction

*Salmonella* infections are the second most frequently reported cause of bacterial gastroenteritis in humans and account for numerous food-borne outbreaks in the European Union and European Economic Area (EU/EEA) [1]. *Salmonella* infections are notifiable in Germany. The mean number of notified cases per year for the period 2015 to 2019 was 13700 (incidence 16.5/100000 population) [2–6]. Probably as a consequence of the pandemic, the number of reported salmonellosis cases decreased by one third in 2020 and 2021 [7]. *Salmonella enterica* subspecies *enterica* serovar (*S.*) Muenchen [6,8:d:1,2] is a less common serovar in Germany with 22–32 reported cases per year in 2015–2019 [8].

According to data from the German National Reference Laboratory for *Salmonella* (NRL) at the German Federal Institute for Risk Assessment (BfR), the serovar Muenchen is among the top 10 of serovars isolated from reptiles in Germany (personal communication with Jennie Fischer; June 2024) [9]. However, also food-producing animals are associated with *S.* Muenchen, in particular pigs [10–12]. A large *S.* Muenchen outbreak in Germany in 2013 and 2014 was linked to consumption of various raw pork sausages. In this outbreak, investigations pointed to the primary production of pigs as the source [12]. In 1999 and 2016, outbreaks with *S.* Muenchen in the United States were linked to alfalfa sprouts from one contaminated seed lot [13, 14]. In Australia, consumption of sea turtles was the cause of an outbreak in a remote Aboriginal community [15]. Unpasteurized orange juice has also been identified as an outbreak vehicle for *S.* Muenchen [16].

In September 2020, the German National Reference Centre for *Salmonella* and other Bacterial Enteric Pathogens (NRC) at the Robert Koch Institute (RKI) observed an unexpected increase in the number of *S.* Muenchen patient isolates. The isolates were primarily from two non-neighbouring federal states in the west and south of Germany. Concurrently, an increase in *S.* Muenchen infections reported to RKI compared with previous years was observed: 52 infections in September 2020 versus a median of 8 infections in September in the years 2015–2019. In the time period January to August 2020, only six infections were reported. Germany shared a reference sequence of the clinical outbreak strain using the European surveillance portal for infectious diseases (EpiPulse).

We conducted an outbreak investigation in order to identify the vehicle of infection and to stop the outbreak.

## Methods

### Data sources

In Germany, laboratory detection of *Salmonella* spp. in clinical specimens is notifiable. Information on acute salmonellosis is reported via the local and subsequent the state health departments to the national surveillance system at RKI.

Regional laboratories voluntarily provide about 4000–5000 clinical *Salmonella* isolates annually to the NRC. The National Reference Laboratory (NRL) receives about 3000 *Salmonella* isolates per year for typing originating from various non-human matrices from all federal states in Germany.

### Definition of WGS clusters, outbreaks, and matches with food and animal isolates

RKI developed an *in-house* definition for *Salmonella* Whole Genome Sequencing (WGS) clusters that require further epidemiological assessment based on core genome Multilocus Sequence Typing (cgMLST).

- A cluster consists of a main node comprising at least four isolates with 0 allele differences (AD) to each other.
- Isolates with 1–3 AD to the main node are also assigned to the WGS cluster.

We classified this WGS cluster as an outbreak because further epidemiological criteria such as temporal clustering and exceeding of the expected number of infections were fulfilled. Read files from representative clinical outbreak strains are regularly provided by the NRC (RKI) to the NRL (BfR).

### Outbreak case definition

Probable cases were defined as patients with laboratory-confirmed *S.* Muenchen infections reported to the national surveillance system or whose *S.* Muenchen isolates were sent to the NRC or federal state laboratories between 1 September 2020 and 31 July 2021. Cases were classified as confirmed if their isolates were assigned to the outbreak WGS cluster or if they had an epidemiological link to a confirmed case. Cases were excluded if 1) their sequenced isolates could not be assigned to the outbreak WGS cluster or 2) if they had travelled outside of Germany in the seven days prior to symptom onset and neither sequence data were available nor an epidemiological link to a confirmed case.

### Descriptive epidemiology

We describe case characteristics by age, sex, place of living, severity of infection, and symptom onset. If symptom onset was unknown, it was estimated from the notification date or the laboratory date (the date the isolate is registered at the NRC) by subtracting one week from date of notification or laboratory date. A map showing the number of cases per 100000 inhabitants by municipality was created using Regiograph.

### Laboratory investigations of clinical isolates

Human clinical isolates sent to the NRC were serotyped according to the Kauffmann–White–LeMinor scheme. Genomic DNA was prepared using the GenElute™ Bacterial Genomic DNA Kit from Sigma-Aldrich according to the manufacturer’s instructions, sent to the RKI sequencing core unit for library preparation (Illumina Nextera XT DNA Library Preparation Kit), and subjected to short-read sequencing (Illumina NextSeq, v3 chemistry, 2 × 150 bp paired end mode). Sequence data from additional clinical *S.* Muenchen isolates were provided by the Institute for Hygiene and Environment in Hamburg and the Bavarian State Office for Health and Food Safety (LGL).

### Laboratory investigations of food and animal isolates

Sequencing analyses of food and veterinary *S.* Muenchen isolates were performed at the NRL, the Institute for Hygiene and Environment in Hamburg, and the Chemical and Veterinary Investigation Office Stuttgart (CVUA-S), Baden Wuerttemberg. Genomic DNA was prepared using the PureLink Genomic DNA Kit from Thermo Fisher Scientific or the Qiagen DNeasy Blood & Tissue Kit according to the manufacturer’s instructions. For the library preparation, the Nextera DNA Flex Library Preparation Kit was used. For the short-read sequencing, the Illumina iSeq, MiSeq or NextSeq500 v3 chemistry, 2 × 151 bp or 2 × 149 bp or NovaSeq 6000, 2 × 100 bp paired end mode were used. Pre-screening analyses were carried out with *in-house* WGS pipelines (AQUAMIS and chewieSnake) at the NRL based on cgMLST comparative analysis using a human clinical reference sequence provided by the NRC [17, 18].

Read files with raw data were then exchanged in accordance with data protection requirements between institutes and at the NRC and imported into Ridom SeqSphere^+^ [19] for subsequent read quality control, de novo assembly, and cgMLST applying the 3002 loci comprising EnteroBase scheme [20, 21]. Genome data from representative clinical outbreak strains and food/animal isolates were uploaded to ENA or NCBI, respectively (ENA project no. PRJEB65349 and NCBI project no. PRJNA1029486, respectively).

### Hypothesis-generating interviews

Cases were interviewed using a hypothesis-generating questionnaire including about 400 questions focusing on food consumption three days before symptom onset. Answer categories for food exposures included: ‘yes’, ‘probably yes’, ‘probably no’, and ‘no’. Interviews were conducted via telephone and answers were transferred into an RKI *in-house* Excel file to identify high portions of similar exposure or other similarities. Proportion of food exposures was compared with results from a 2017 national food consumption survey [22].

### Case–control study

A case–control study was conducted in the two most affected federal states between 29 January and 12 February 2021 to test the hypothesis of dried coconut pieces being the vehicle of infection. Cases older than 18 years residing in Bavaria or North Rhine-Westphalia for whom contact details were available were included. Controls were recruited and interviewed by a market and social research institute using random digit dialling of landline numbers. Per case, four suitable controls, frequency-matched by age group (18–35, 36–55, 56–75 years) and sex within each federal state of residence, were selected. To ensure equal exposure opportunity, controls were recruited from municipalities with stores of a certain retail chain mentioned notably often in the hypothesis-generating interviews in which the coconut pieces under suspicion had been sold. Controls with symptoms of an acute gastrointestinal infection or who had travelled outside their federal state within the three days before the interview were excluded. All interviews of controls were conducted using computer-assisted telephone interviewing (CATI). Exposure period was defined as the three-day time period prior to disease onset (cases) or the interview (controls).

We compared the frequency of consumption of queried food items between cases and controls. Cases and controls were considered exposed if they answered ‘yes’ or ‘probably yes’. The Chi-square test for homogeneity was used to calculate differences in frequency distributions for place of living (federal state), sex, and age groups between cases of the case–control study and the controls. Cases reporting coconut product consumption were asked if they could recall the consumed product among eight different coconut products (pictures).

We used logistic regression to calculate adjusted odds ratios (aOR) with 95% confidence intervals (CI). The OR for being a case having consumed a certain food item was adjusted for federal state of residence, age group, and sex. In multivariable analysis, aOR for being a case when having eaten dried coconut pieces were further adjusted for other consumed food items if the respective odds of being a case was higher than for controls (lower bound of the 95% CI >1). Sensitivity analysis was conducted looking only at cases and controls having replied ‘yes’. Analyses were conducted using the statistics software Stata (version 17.0, Stata Corporation, USA).

### Food investigations and traceback

The local food safety authorities performed food investigations and traceback. Information regarding food traceback and production details was forwarded via the state authorities for food safety to the BVL, which then shared the results with the health sector. Open packages of dried coconut pieces were sampled from households of three cases as part of the outbreak investigation additional to routine testing. The samples were analysed at the official food safety laboratories.

## Results

**Figure 1.**
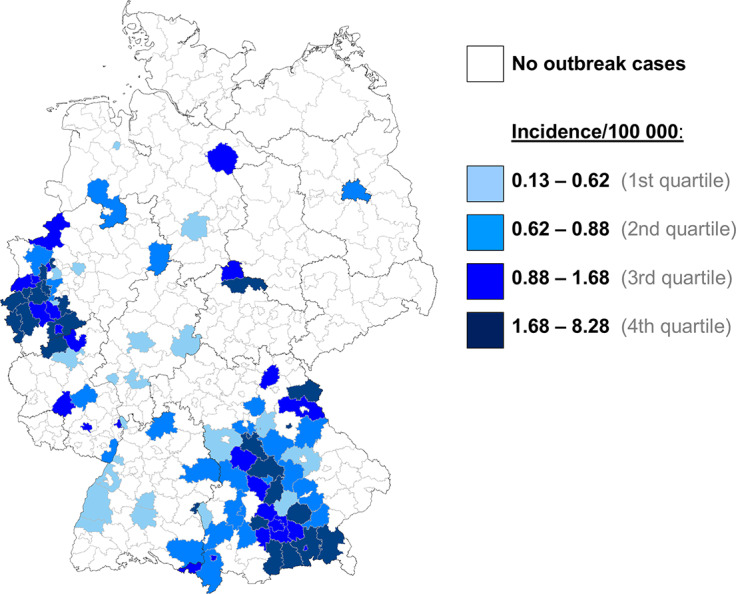
Number of *S.* Muenchen outbreak cases per 100,000 inhabitants by municipality, Germany, 2019–2020.

In total, 301 outbreak cases were identified, of which 129 were confirmed. There were no cases reported from outside of Germany. Most cases (251/301 cases; 83%) lived in two non-neighbouring federal states in the west and in the south of Germany: North Rhine-Westphalia (*n* = 143) and Bavaria (*n* = 108). The remaining cases lived in six other federal states across Germany (3–16 cases per state) (Figure 1 and Supplementary Material S1). The majority of the cases was female (206/301; 68%), and the median age was 49 years (range 0–100 years; interquartile range 30–60 years). For 186 cases (62%), complete information regarding hospitalization was reported. Of those, 31 cases needed to be hospitalized due to salmonellosis (17%). The median duration of hospitalization was four days (range 1–15 days). No deaths were reported.

**Figure 2.**
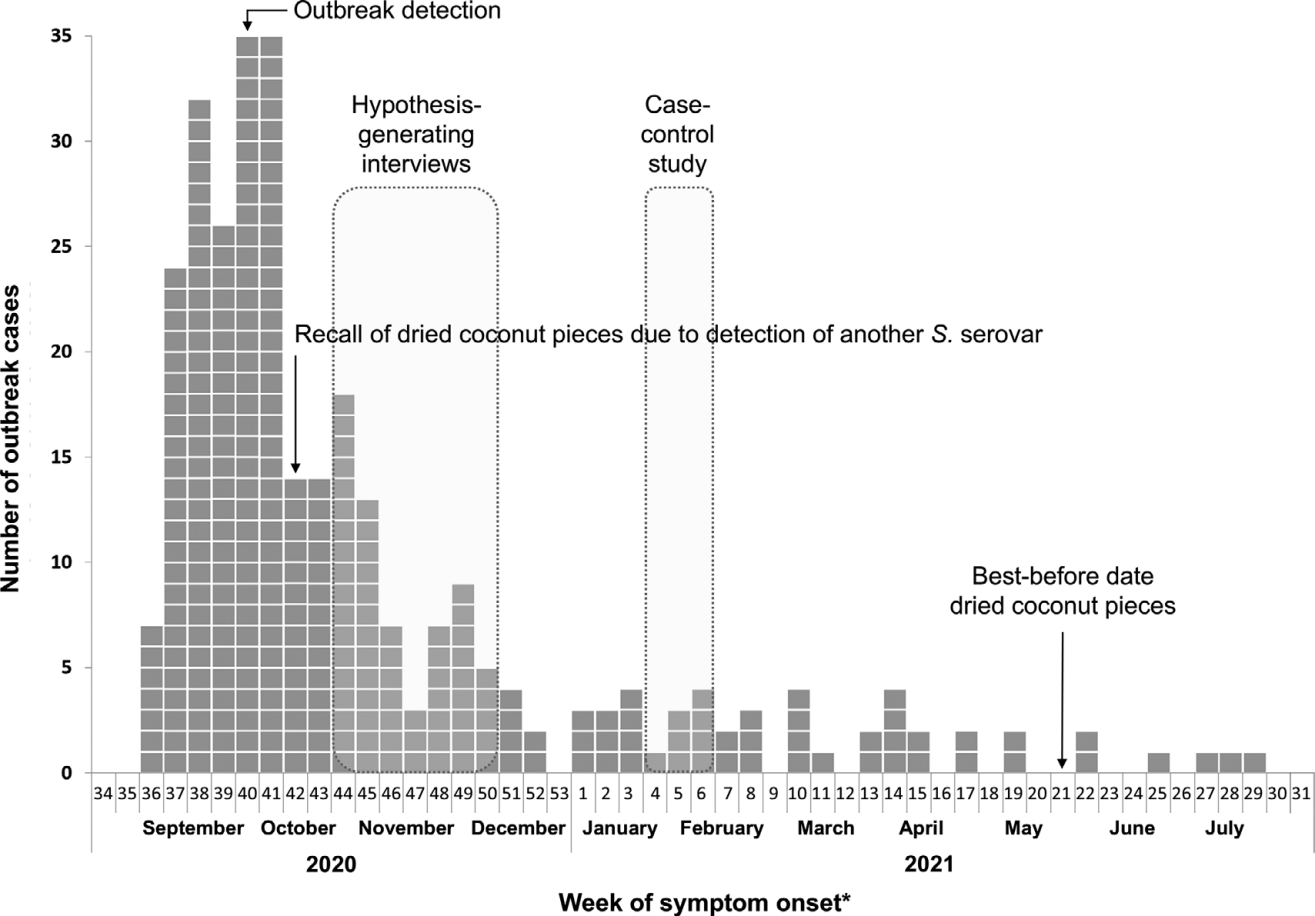
Chronology of the outbreak: Number of outbreak cases by week of symptom onset: 2020 to 2021, Germany (*n = 301*). *If the week of symptom onset was unknown, the week before notification or arrival of the isolate at the NRC was used.

Symptom onset was reported for 153/301 (51%) cases from 2 September 2020 to 5 July 2021. For the remaining cases, symptom onset was estimated. About half of the cases (152/301) had symptom onset in the calendar weeks 37–41 of 2020 (September to mid-October 2020). Thereafter, the weekly number of cases decreased and stayed at a low level until calendar week 29 in 2021 (end of July 2021) (Figure 2).

### Sequence analysis

In the course of this outbreak investigation, 139 clinical *S.* Muenchen isolates were sequenced and analysed with 129 (93%) belonging to the outbreak WGS cluster. Of the 129 clinical isolates, 116 belonged to the main node of the WGS cluster with 0 AD, and 13 showed 1 to 2 AD to the main node and a maximum of 3 AD to each other. Representative genome sequences are available in ENA under project number PRJEB65349. In NCBI, the genome data from swan, milk powder, and coconut products are available in the project PRJNA1029486 (Biosamples SAMN37871446, SAMN37871445, SAMN37939464–SAMN37939468).

**Figure 3.**
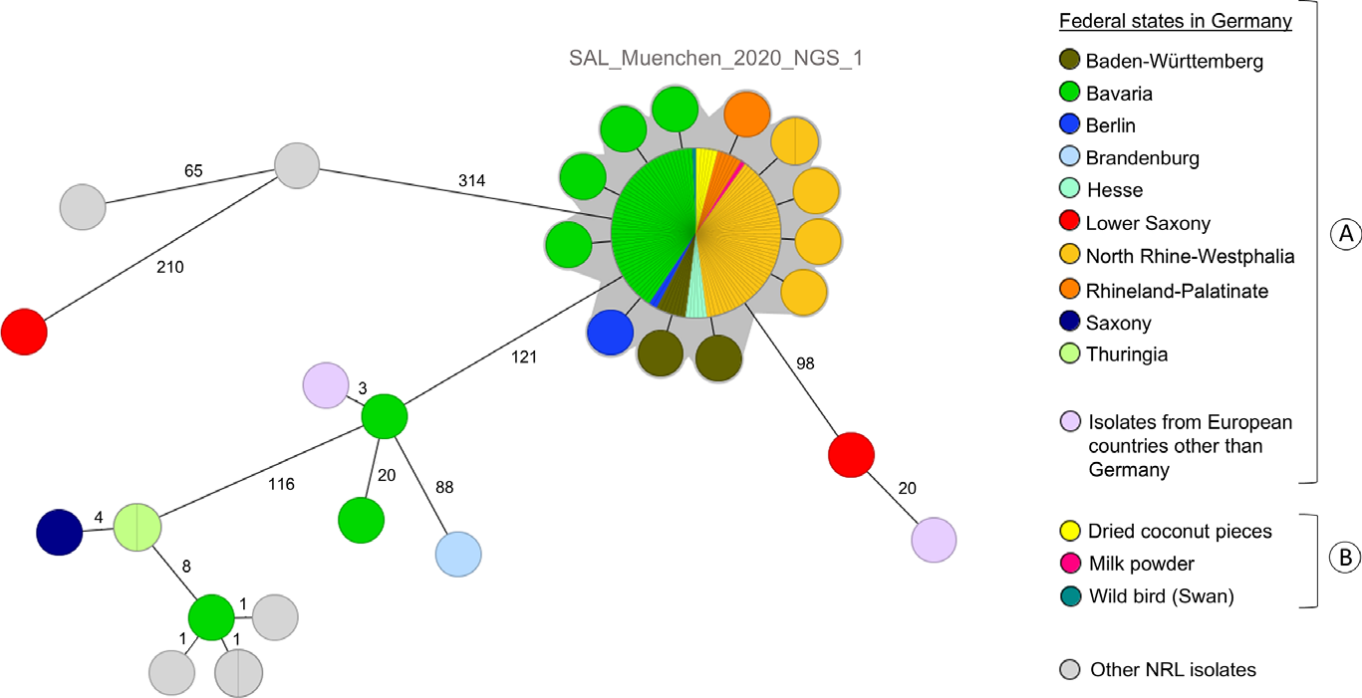
Minimum Spanning Tree based on the EnteroBase cgMLST scheme comprising 3002 loci created with Ridom SeqSphere^+^ (pairwise, ignoring missing values) including [A] clinical and [B] food and animal isolates.

Other sequenced *S.* Muenchen clinical isolates showed at least 98 AD to the main node and were excluded from the outbreak (Figure 3).

### Food and animal isolates

In total, six isolates and/or sequence data sets originating from food (coconut products (*n = 5*) and milk powder (*n = 1*)) were sent to the NRL during the outbreak investigation. These isolates originated from company’s own quality control checks or were taken due to consumer complaints. Another 16 *S.* Muenchen isolates from the NRL strain collection received in 2020 from reptiles (6), spices (2), fertilizers (2), feed (1), meat (1), and wild bird (swan) (1) and in 2019 from unknown sources others than reptiles (3) were subjected to WGS for cgMLST analysis and comparison with the outbreak reference strain.

The NRL identified seven isolate sequence matches with the human outbreak strain from three different matrices: one from milk powder used for chocolate production (sent to the laboratory in October 2020), five from dried coconut pieces (sampled in September/October and December 2020), and one from a wild swan (sampled in November 2020). The NRC confirmed 0 AD between these sequences and the main node of the outbreak WGS cluster (Figure 3). Three additional opened packages of dried coconut pieces were sampled in three patients’ households. These products tested negative.

### Hypothesis-generating interviews

From 28 October to 11 December 2020, 36 cases participated in hypothesis-generating interviews. No clear hypothesis for a vehicle of infection could be generated. However, 83% (30/36) of cases often bought their food at one retail chain. In comparison, this chain was mentioned by 53% in a national food consumption survey in the adult population from 2017 [22]. The most frequently mentioned consumed food items were pasta (28/34; 82%), tea (27/35; 77%), cucumber (26/34; 76%), and apples (24/34; 71%). Some food items (apples, cucumbers) are known to be frequently consumed by the general adult population in Germany [22]. Consumption of coconut pieces was remembered by 16/34 (47%) cases and consumption of chocolate by 15/34 (44%) cases. No specific brand of food items was mentioned more frequently than others.

### Case–control study

Out of the 301 identified cases in the outbreak, 30 could be included in the case–control study; 26 of the 36 cases that had been interviewed for hypothesis generation and four cases not previously interviewed. In total, 116 controls were recruited. Compared with all cases, cases included in the case–control study showed a similar age and sex distribution (Supplementary Material S1).

Comparing the cases included in the study with the controls, the distribution of cases and controls within most of the age groups did not differ significantly. However, differences were apparent in the 18–35 years age group (12/30 cases (40%) vs. 26/116 controls (22%)) (Supplementary Material S1).

Of 30 cases in the case–control study, 22 (73%) reported having eaten dried coconut pieces. In contrast, only two of the 116 controls (2%) had eaten these products (aOR 176; 95% CI: 32–954, *p* < 0.001). Cases also reported consuming coconut milk, grated coconut (rasp), warm meals with coconut, ice cream, and Gouda cheese more frequently than controls. However, the aOR were substantially smaller than for coconut pieces (Table 1; a table with all exposures can be found in the Supplementary Material S2). Consumption of dried coconut pieces remained the only food item significantly associated with being a case in multivariable models adjusted for consumption of the other food items (Supplementary Material S3). The sensitivity analysis showed no significant changes in OR for exposure of dried coconut pieces (data not shown). Cases to whom photos of different brands of commercially available dried coconut products were presented, largely recognized (15/27 cases (56%)) the brand of dried coconut pieces in which the outbreak strain had been detected.

**Table 1.** Consumption of food items by cases and controls and OR adjusted for matching variables (a table with all exposures included can be found in the Supplementary Material S2)

Exposure	Cases			Controls			aOR^b^	95% CI	*p-value*
	*N* exposed^a^ %			*N* exposed^a^ %					
**Dried coconut pieces**	**30**	**22**	**73**	**116**	**2**	**2**	**176**	**32–954**	***<0.001***
Coconut milk	30	9	30	116	5	4	**8.8**	2.5–30	*0.001*
Grated coconut (rasp)	30	10	33	116	8	7	**6.3**	2.1–18	*0.001*
Warm meal with coconut	30	7	23	114	6	5	**5.8**	1.7–20	*0.005*
Ice cream	29	15	52	114	22	19	**4.4**	1.8–11	*0.001*
Gouda cheese	26	20	77	116	52	45	**3.5**	1.2–9.7	*0.02*
Coconut cakes/cookies	30	2	7	114	3	3	**3.3**	0.5–23	*0.23*
Muesli	30	15	50	116	33	28	**2.4**	1.0–5.6	*0.05*
Yoghurt	26	17	65	116	64	55	**1.9**	0.7–4.6	*0.19*
Desserts with coconut	30	1	3	114	2	2	**1.8**	0.1–22	*0.65*
Milk	27	21	78	116	88	76	**1.2**	0.4–3.4	*0.71*
Any chocolate products^c^	30	20	67	114	72	63	**1.0**	0.4–2.4	*0.96*

a Exposed = cases/controls who answered ‘yes’ or ‘probably yes’ to consumption of the respective product in the three days prior illness/interview.

b aOR, odds ratio adjusted for age group, sex, and state of residence.

c Containing chocolate mousse, chocolate pudding, chocolate tablet, chocolate bar, couverture chocolate, and/or chocolate with coconut.

### Food investigations and traceback

The following traceback information was available for the different samples from which the outbreak strain was isolated:

1. *Milk powder.* The milk powder was produced on 8 October 2020 (calendar week 41) from pasteurized milk obtained from different dairy farmers in the region. After being tested positive for S*almonella* during one of the company’s own quality control checks, the milk powder was liquefied, pasteurized, and dried again, whereupon *Salmonella* could not be detected anymore. It remained unclear, at which production step S*almonella* contamination of the product had occurred. The then twice-pasteurized milk powder was processed by other companies exclusively for chocolate production. Detailed information on the kinds of chocolate products the milk powder was used for was not available.
2. *Dried coconut pieces.* In total, seven samples of dried coconut pieces of the same company tested positive for *S.* Muenchen: 1× company’s own quality control check sampled in September 2020, 2× official controls from December 2020, and 4× consumer complaints. Five of the seven isolates were sequenced and belonged to the main node of the outbreak cluster with 0 AD (Figure 3). The dried coconut pieces of a specific lot tested also positive for *Salmonella* subspecies II (*salamae*) during one of a company’s own quality control checks. This led to a product recall by the company and a public warning on 14 October 2020.
  Food traceback of the dried coconut pieces led to four different countries of origin (Mozambique, Ghana, Sri Lanka, and Burkina Faso) that had sent dried coconut pieces to the same packaging company in Türkiye. However, the lot that tested positive for *Salmonella* originated only from Mozambique. The ready-for-consumer-use product had been transported from Türkiye to a logistics company in the south of Germany, which initiated the company’s own quality control checks. The product was exclusively sold in Germany by a common retail chain. Notably, this was the same retail chain which was mentioned most often in the explorative interviews. The implicated lot had a best-before date of 24 May 2021.
3. *Swan.* A sample from a dead wild swan found near a sewage treatment plant was investigated by the responsible veterinary authority and tested positive for a strain of *S.* Muenchen that belonged to the main node of the outbreak cluster with 0 AD. We were unable to establish how the swan had become infected.

### Outbreak control measures

Due to detection of *Salmonella* subspecies II (salamae) during a company’s own quality control check, a product recall of the dried coconut pieces was already initiated before the first case interviews were conducted. At that time of recall, a connection with the outbreak was not obvious. Only later, *S.* Muenchen was also detected in the same product. After the recall, a remarkable decrease in case numbers is visible (Figure 2). However, due to the long shelf-life of the product and possibly not recognizing the recall, cases occurred up to 9 months after recall.

## Discussion

As a result of an efficient collaboration between epidemiology, microbiology, and food safety agencies, dried coconut pieces were linked to this large outbreak in Germany. This outbreak investigation demonstrated the importance of close inter-sectoral cooperation and the power of timely routine sharing of reference genome sequences from patient isolates, food, and animal isolates. There were three different matrices from which the outbreak strain was isolated: milk powder, dried coconut pieces, and a wild animal (swan). A connection between these three matrices was not apparent. There are multiple possible links between the clinical outbreak strain and the identified isolates from food and the wild swan. Waste water or asymptomatic patients shedding *Salmonella* might be potential sources.

In this outbreak investigation, the molecular matches were the prerequisite for conducting a targeted epidemiological analytical study (case–control study). The case–control study then showed a strong association between consumption of dried coconut pieces and disease. As it was unknown what chocolate products were produced using the dried milk powder, we were not able to test a hypothesis for specific chocolate products in an analytical study. However, looking at general chocolate consumption, the odds of being a case was not statistically different from being a control in the conducted case–control study. The association between having consumed dried coconut pieces and being a case may be overestimated, as we conducted the study in January/February, whereas consumption of coconut products in the general population may have been higher in the months before Christmas. Another possible reason for potential of overestimation could be that some of the cases who were interviewed for the case–control study had previously been interviewed in the hypothesis-generating interviews. However, the extremely high adjusted odds ratio, the recognition of the product by cases presented with pictures of different coconut products, the WGS match between clinical isolates and the coconut isolate, and the plausible traceback investigations indicate that the dried coconut pieces constitute the most likely vehicle of infection in this outbreak. In addition, the long shelf-life of the coconut pieces may explain the long duration of this outbreak.

In the 1950s and 1960s, *Salmonella* in dried coconut pieces was a well-recognized problem [23–25], and outbreaks were reported (e.g. in Liverpool, England, 1960–1961) [26]. More recently, only two *Salmonella* outbreaks have been linked to consumption of dried coconut pieces: one outbreak was caused by *S.* Chailey in the United States and Canada in 2017 [27]; the second outbreak occurred in the United States in 2018 and was caused by *S.* Typhimurium [28]. Contamination with *Salmonella* likely happens by contact with soil contaminated by faecal matter, when the coconuts detached from the trees are stored on the ground [26]. Once bacteria enter through small cracks in the nut shell, the bacteria may find optimal conditions for multiplying inside the coconut without causing the coconut to show any signs of deterioration. During the following processing steps, the contamination can persist and spread further [26]. This may be a plausible explanation for the outbreak scale. In 1967, Schaffner and colleagues described a pasteurization step in the processing of dried coconut pieces, which was effective in terms of decontamination without impairing the quality of the product. The authors state that this method has since been widely used in the coconut industry [26]. In fact, for several decades after that publication in 1967, dried coconut pieces were not reported in the published literature as vehicles of infection for *Salmonella* outbreaks. If not done so already, an obligatory pasteurization step should be required in the processing of dried coconut.

The focus of this outbreak investigation was set on the genetically linked isolates from the two different potential food sources. Epidemiological studies (e.g. case–control studies) are necessary for the confirmation of hypotheses. However, it was striking that all three samples originated from the outbreak period and (at least the milk powder and the dried coconut pieces) from the two federal states where most cases were reported. The question remains whether there was really no connection between the samples from three different matrices or if investigations were not sufficiently detailed to disclose it. For most food-borne outbreaks, the potential point of contamination in the food chain is not determined. However, the information about where and when the contamination has occurred may be key for developing more effective prevention strategies.

This inter-sectoral outbreak investigation emphasizes that a genome-based match alone cannot be regarded as sufficient evidence to declare a respective food item as the vehicle of infection. Further information is needed through epidemiological and possibly from other, e.g. environmental or traceback investigations. All available evidence has to be considered and reviewed continuously by the outbreak investigation team to allow identification of the most likely vehicle of infection.

## Supporting information

10.1017/S0950268826101423.sm001 Staat et al. supplementary material Staat et al. supplementary material

## Data Availability

Representative genome sequences are available in ENA under project number PRJEB65349. In NCBI the genome data from swan, milk powder and coconut products are available in the project PRJNA1029486 (Biosamples SAMN37871446, SAMN37871445, SAMN37939464–SAMN37939468).

## Supplementary material

The supplementary material for this article can be found at http://doi.org/10.1017/S0950268826101423.
