# Supplementary material for: Large outbreak of Salmonella Muenchen linked to dried coconut pieces, September 2020 to July 2021, Germany
Source: Epidemiol Infect. 2026 Apr 17;154:e55. doi: 10.1017/S0950268826101423 (PMC13161823; doi:10.1017/S0950268826101423)
Supplement: Staat et al. supplementary material [file S0950268826101423sup001.docx]

**Large outbreak of *Salmonella* Muenchen linked to dried coconut pieces, September 2020 to July 2021, Germany**

Staat, Doreen^#^; Simon, Sandra^#^; Pietsch, Michael; Lamparter, Marina C.; Fischer, Jennie; Schewe, Thomas; Rosner, Bettina M.; Wagner, Tobias, Siling, Katja; Overhoff, Judith; Schäfers, Christian; Aust, Olivier; Hiller, Ekkehard; Flieger, Antje; Stark, Klaus, Gillesberg Lassen, Sofie^#^; Meinen, Anika^#^

^#^ These authors contributed equally to the work of the article and share first/last authorship.

*Please see main text file for affiliation list.*

**Corresponding author**: Anika Meinen ([meinena@rki.de](mailto:meinena@rki.de))

## Supplementary Material

**Supplementary Table S1:** Characteristics of the outbreak cases and case-control study population.

|  | | | | | | | | | **Case-control study** | | | | ***p*-value** | |
| --- | --- | --- | --- | --- | --- | --- | --- | --- | --- | --- | --- | --- | --- | --- |
|  | | | | **Outbreak cases** | | | | | **Cases** | | **Controls** | |  |  |
|  | | | | | | (N=301) | | (N=30) | | | (N=116) | |  |  |
| **Residence** | | | |  | | | | | | | | 0.99 ^a, b^ | | |
| NRW | | | n (%) | | 143 (48) | | | | 21 (70) | 81 (70) | | |  | |
| Bavaria | | | n (%) | | 108 (36) | | | | 9 (30) | 35 (30) | | |  | |
| BW | | | n (%) | | 16 (5) | | | | - | - | | |  | |
| RP | | | n (%) | | 14 (5) | | | | - | - | | |  | |
| Hesse | | | n (%) | | 6 (2) | | | | - | - | | |  | |
| Lower Saxony | | | n (%) | | 6 (2) | | | | - | - | | |  | |
| Thuringia | | | n (%) | | 3 (1) | | | | - | - | | |  | |
| Berlin | | | n (%) | | 3 (1) | | | | - | - | | |  | |
| unknown | | | n (%) | | 2 (1) | | | | - | - | | |  | |
| **Sex** | | |  | |  | | | |  | 0.58 ^a, b^ | | | | |
| Female | | | n (%) | | 206 (68) | | | | 22 (73) | 79 (68) | | |  | |
| Male | | | n (%) | | 95 (32) | | | | 8 (27) | 37 (32) | | |  | |
|  |  | |  | |  | | | |  |  | | |  | |
| **Age** | | Median (min, max) | | | 49  (0, 100) | | | | 50  (18, 73) | 51 ^c^  (18, 75) | | |  | |
| **Age groups** | | | |  | | |  | |  | 0.14 ^a, b^ | | | | |
| < 18 y | | | n (%) | | 44 (15) | | | | - | - | | |  | |
| 18 - 35 y | | | n (%) | | 62 (21) | | | | 12 (40) | 26 (22) | | |  | |
| 36 - 55 y | | | n (%) | | 93 (31) | | | | 8 (27) | 45 (39) | | |  | |
| 56 - 75 y | | | n (%) | | 73 (24) | | | | 10 (33) | 45 (39) | | |  | |
| > 75 y | | | n (%) | | 28 ( 9) | | | | - | - | | |  | |
| unknown | | | n (%) | | 1 ( 0) | | | | - | - | | |  | |

NRW=North Rhine-Westphalia, BW=Baden-Württemberg, RP=Rhineland-Palatinate

^a^ chi-square test for homogeneity

^b^ comparison of frequency distributions between cases included in the case-control study and controls

^c^ N=114; reduced due to missing information

Due to rounding, the total may not add up to 100%.

**Supplementary Table S2:** Consumption of all food items by cases and controls in the case-control study.

| **Exposure** | **Cases** | | | |  | **Controls** | | | **aOR^b^** | **95% CI** | | ***p*-value** |
| --- | --- | --- | --- | --- | --- | --- | --- | --- | --- | --- | --- | --- |
|  | **N exposed**^a^ **%** | | | | **N exposed**^a^ **%** | | | |  |  |  |  |
| **Products with coconut** | |  |  |  |  | |  |  |  |  |  | |
| Dried coconut pieces^c^ | | 30 | 22 | 73 | 116 | | 2 | 2 | **176** | **32 - 954** | **<0.001** | |
| Coconut milk | | 30 | 9 | 30 | 116 | | 5 | 4 | **8.8** | **2.5 - 30** | **0.001** | |
| Grated coconut (rasp) | | 30 | 10 | 33 | 116 | | 8 | 7 | **6.3** | **2.1 - 18** | **0.001** | |
| Warm meal with  coconut | | 30 | 7 | 23 | 114 | | 6 | 5 | **5.8** | **1.7 - 20** | | **0.005** |
| Coconut cakes/ cookies | | 30 | 2 | 7 | 114 | | 3 | 3 | **3.3** | 0.5 - 23 | | 0.23 |
| Chocolate with coconut | | 30 | 2 | 7 | 114 | | 3 | 3 | **3.1** | 0.5 - 21 | | 0.25 |
| Desserts with coconut | | 30 | 1 | 3 | 114 | | 2 | 2 | **1.8** | 0.1 - 22 | | 0.65 |
| Candy with coconut | | 30 | 1 | 3 | 114 | | 5 | 4 | 0.6 | 0.1 - 5.2 | | 0.61 |
| Dried fruit with coconut | | 30 | 5 | 17 | 116 | | 0 | 0 | - | - | | - |
| Rawbar with coconut | | 29 | 0 | 0 | 114 | | 0 | 0 | - | - | | - |
| Coconut “flour” | | 30 | 0 | 0 | 116 | | 0 | 0 | - | - | | - |
| Salat with coconut | | 30 | 0 | 0 | 114 | | 0 | 0 | - | - | | - |
| **Muesli** | | 30 | 15 | 50 | 116 | | 33 | 28 | **2.4** | 1.0 - 5.6 | | 0.05 |
| **Milk products** | |  |  |  |  | |  |  |  |  | |  |
| Ice cream | | 29 | 15 | 52 | 114 | | 22 | 19 | **4.4** | **1.8 - 11** | | **0.001** |
| Gouda cheese | | 26 | 20 | 77 | 116 | | 52 | 45 | **3.5** | **1.2 - 9.7** | | **0.02** |
| Yoghurt | | 26 | 17 | 65 | 116 | | 64 | 55 | **1.9** | 0.7 - 4.6 | | 0.19 |
| Milk | | 27 | 21 | 78 | 116 | | 88 | 76 | **1.2** | 0.4 - 3.4 | | 0.71 |
| Butter | | 26 | 17 | 65 | 116 | | 84 | 72 | 0.8 | 0.3 - 2.1 | | 0.65 |
| Soft cheese | | 26 | 9 | 35 | 116 | | 42 | 36 | 0.8 | 0.3 - 2.0 | | 0.64 |
| Quark | | 26 | 7 | 27 | 116 | | 41 | 35 | 0.6 | 0.2 - 1.7 | | 0.36 |
| **Chocolate products** | |  |  |  |  | |  |  |  |  | |  |
| Chocolate mousse | | 30 | 3 | 10 | 116 | | 3 | 3 | **3.5** | 0.6 - 19 | | 0.15 |
| Chocolate pudding | | 30 | 5 | 17 | 116 | | 6 | 5 | **2.9** | 0.8 - 11 | | 0.12 |
| Couverture chocolate | | 30 | 3 | 10 | 116 | | 6 | 5 | **1.5** | 0.3 - 6.7 | | 0.66 |
| Chocolate tablet | | 30 | 13 | 43 | 116 | | 46 | 40 | **1.1** | 0.5 - 2.6 | | 0.79 |
| Chocolate bar | | 30 | 8 | 27 | 116 | | 36 | 31 | 0.6 | 0.2 - 1.7 | | 0.37 |
| Chocolate sauce | | 30 | 0 | 0 | 116 | | 0 | 0 | - | - | | - |

^a^ exposed = cases/controls who answered “yes” or “probably yes” to consumption of the respective product in the three days prior illness/interview

^b^ aOR: Odds ratio adjusted for age group, sex and state of residence

^c^ includes dried coconut pieces and/or dried coconut chips

**Supplementary Table S3:** Multivariable models with consumption of dried coconut pieces adjusted for consumption of other food items (case-control study).

| **Models** | **Dried coconut pieces ^a,b^** | | |  | **Respective food item ^c^** | | |
| --- | --- | --- | --- | --- | --- | --- | --- |
|  | **aOR** | **95% CI** | ***p*-value** |  | **aOR** | **95% CI** | ***p*-value** |
| **A:** Dried coconut pieces ^a,b^ & Coconut milk **^c^** | **148** | **26-884** | **<0.001** |  | 1.9 | 0.2-17 | 0.567 |
| **B:** Dried coconut pieces ^a,b^ & Warm meal with coconut **^c^** | **158** | **28-897** | **<0.001** |  | 1.5 | 0.2-14 | 0.704 |
| **C:** Dried coconut pieces ^a,b^ & Ice cream **^c^** | **142** | **25-795** | **<0.001** |  | 3.2 | 0.8-12 | 0.090 |
| **D:** Dried coconut pieces ^a,b^ & Gouda cheese **^c^** | **121** | **22-673** | **<0.001** |  | 1.7 | 0.4-7.3 | 0.469 |

^a^ multivariable model was adjusted for age group, sex, state of residence and additionally for another consumed food item where the odds of being a case was higher than for controls (95% CI >1)

^b^ includes dried coconut pieces and/or dried coconut chips

^c^ multivariable model was adjusted for age group, sex, state of residence and additionally for consumed dried coconut pieces
